# The role of Activin A in fibrodysplasia ossificans progressiva: a prominent mediator

**DOI:** 10.1042/BSR20190377

**Published:** 2019-08-02

**Authors:** Hui Lin, Fuli Shi, Jiayu Gao, Ping Hua

**Affiliations:** Jiangxi Province Key Laboratory of Tumor Pathogens and Molecular Pathology and Department of Pathophysiology, School of Basic Medicine Sciences, Nanchang University Medical College, Nanchang, China

**Keywords:** Activin A, ALK2, FOP, Heterotopic ossification

## Abstract

Heterotopic ossification (HO) is the aberrant formation of mature, lamellar bone in nonosseous tissue. Fibrodysplasia ossificans progressiva (FOP) is a rare and devastating genetic disorder that causes progressive HO in the ligaments, tendons, and muscles throughout the body. FOP is attributed to an autosomal mutation in activin receptor-like kinase 2 (ALK2), a bone morphogenetic protein (BMP) type I receptor. Initial studies show that mutant ALK2 drives HO by constitutively activating the BMP signaling pathway. Recently, mutant ALK2 has been shown to transduce Smad1/5 signaling and enhance chondrogenesis, calcification in response to Activin A, which normally signals through Smad2/3 and inhibits BMP signaling pathway. Furthermore, Activin A induces heterotopic bone formation via mutant ALK2, while inhibition of Activin A blocks spontaneous and trauma-induced HO. In this manuscript, we describe the molecular mechanism of the causative gene *ALK2* in FOP, mainly focusing on the prominent role of Activin A in HO. It reveals a potential strategy for prevention and treatment of FOP by inhibition of Activin A. Further studies are needed to explore the cellular and molecular mechanisms of Activin A in FOP in more detail.

## Background

Fibrodysplasia ossificans progressiva (FOP) is a rare and devastating genetic disorder, which is characterized by progressive heterotopic ossification (HO) of muscle, tendon, and ligamentous tissues throughout the body. The ectopic bone formation causes chronic pain, joint ankylosis, and immobilization, which cumulatively lead to premature death due to breathing difficulties [1]. HO formation in FOP may be spontaneous or triggered by trauma(s) and inflammation [2]. The incidence of FOP is approximately one in two million [3]. FOP is caused by gain-of-function mutations in activin receptor-like kinase 2 (*ALK2*, also known as *ACVR1*), a bone morphogenetic protein (BMP) type I receptor [4,5]. It is the first significant discovery in understanding the genetic and molecular mechanisms of HO in FOP.

**Figure 1:**
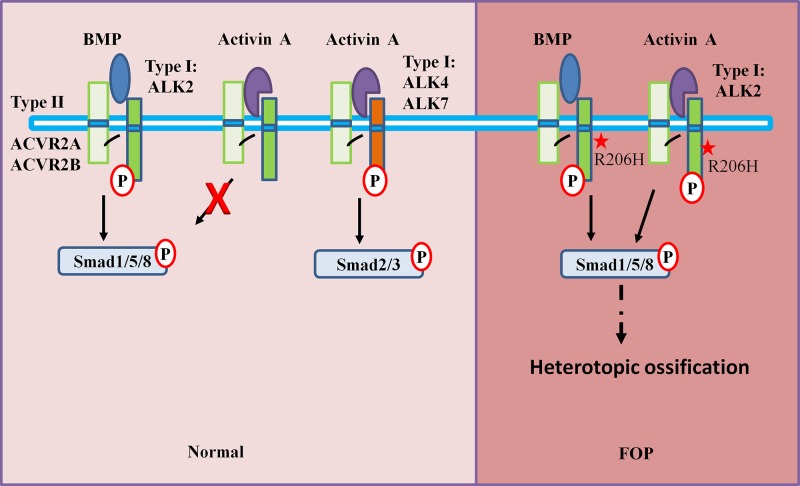
Schematic diagram of the BMP/Activin A signaling network in normal and FOP patients BMP signals via the BMP type I receptor ALK2, inducing phosphorylation of Smad1/5, whereas Activin A induces the phosphorylation of Smad2/3 via ALK4/7. It also binds to ALK2, but it does not activate Smad1/5 phosphorylation and inhibits BMP signaling. BMP and Activin A share type II receptors (ACVR2A, ACVR2B). However, in FOP, Activin A leads to activation of Smad1/5 phosphorylation via mutant ALK2 and triggers HO formation. The star: classic mutation site of ALK2 R206H. Abbreviation: P, phosphorylation.

Initial studies have shown that the mutation in ALK2 confers hyperactivity in response to BMP ligands and constitutive activity in the absence of ligands [6–8]. Recently, studies have shown that Activin A, a ligand that normally transduces Smad2/3 signaling, activates the Smad1/5 signaling pathway through mutant ALK2 [6–8]. Both BMP and Activin A belong to the transforming growth factor-β (TGF-β) family. Based on their biological functions in the induction of bone formation, Activins are nonosteogenic and BMPs are osteogenic [9]. Activin A turns on osteogenic signaling, similar to BMP, and enhances chondrogenesis in FOP cells [10,11]. Subsequently, Activin A has been reported to trigger heterotopic bone formation, while inhibition of Activin A blocks the progression of spontaneous or trauma-induced HO in a mouse model of FOP [10,12]. All of these findings indicated that Activin A is involved in the pathophysiology of HO in FOP (Figure 1). However, the underlying molecular mechanisms driving mutant ALK2 in response to Activin A remain unclear. Currently, there are no effective or specific therapies for FOP. The discovery of *ACVR1/ALK2* gene responsible for FOP unveils a potential therapeutic for FOP. In this review, we briefly recapitulate the role of mutant ALK2 in FOP, paying particular attention to the underlying molecular mechanism driving mutant ALK2 activation by Activin A. Moreover, we review the current research on the prominent role of Activin A in HO and the implications in the treatment of FOP.

## FOP

### Association of ALK2 mutation with FOP

**Figure 2:**
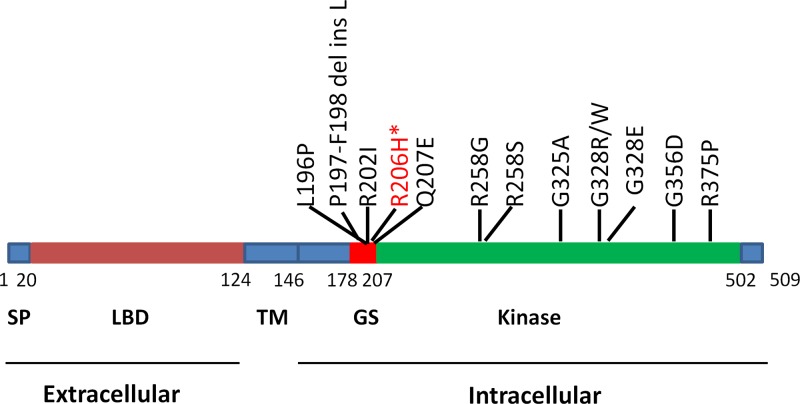
Schematic diagram showing the numbers and locations of ALK2 mutations associated with FOP *R206H is the most common mutation in FOP. Abbreviations: LBD, ligand binding domain; SP, signal peptide; TM, transmembrane.

ALK2 is a highly conserved serine/threonine kinase characterized by functional domains. ALK2 contains an extracellular N-terminal domain for ligand binding, a transmembrane domain, and a cytoplasmic C-terminal kinase domain, which consists of a glycine-serine-rich domain (GS domain) and a protein kinase domain (Figure 2) [13–15]. To date, 13 FOP-associated ALK2 mutations have been identified (Figure 2) [1,5,16–21]. All of the ALK2 mutations occur in the intracellular domain (Figure 2). Most FOP patients (typical FOP, more than 95% of cases) bear a heterozygous mutation in the *ALK2* gene with an arginine to histidine substitution at position 206 (R206H) (Figure 2) [4,10,22]. The phenotype of FOP patients appears to depend on the variations in ALK2 mutations. For typical mutation of ALK2, in addition to progressive HO in extraskeletal sites, typical FOP patients show clinical features, with great toe malformation [5]. Postnatal HO in FOP usually begins in childhood [23,24]. However, there is phenotype variability among individuals carrying the same mutation, such as the age of onset of HO and rate of HO progression [19]. Additionally, patients harboring non-R206H ALK2 mutations display variability in clinical features. For example, patients with p.L196P have delayed-onset HO and normal toes but have a mild clinical course, with fifth-finger bilateral camptodactyly [18]. Very-early-onset HO was found in a patient with G328W or G328E, and multiple digits and thumb deformities have been reduced [19]. However, these studies did not determine how and why the onset of HO in FOP is associated with different mutations. Previous studies indicated that delayed onset of HO might be related to the specific domain of the ALK2 mutation [25]. Nevertheless, other studies show that nongenetic factors (environmental factors) are strongly involved in HO onset and progression in monozygotic twins with FOP [1]. Further functional studies are needed to elucidate clinical presentation correlations with various ALK2 mutations.

### ALK2 signaling in FOP and heterotopic bone formation

Many studies have demonstrated that all mutations in ALK2 are gain-of-function mutations and transduce overactive ALK2 signaling [2,26–28]. Mutant ALK2 proteins acquire the capacity for hypersensitivity in response to BMP ligands, as well as enhance the basal receptor activity. Classical FOP has gained the majority of exciting investigations of the molecular mechanisms underlying mutant ALK2. The R206H amino acid substitution leads to a conformational change in ALK2, which exhibits loss of auto-inhibition of the receptor and reduces the binding of ALK2 to FK506-binding protein 1A (FKBP12), the inhibitory protein of ALK2 that generally binds the GS domain [4,29–32]. Thus, the mutated ALK2 is overactivated, which then enhances downstream signaling cascades, including canonical Smad and p38 mitogen-activated protein kinase (MAPK) signaling [7,33,34]. However, studies have shown that the BMP type II receptor is still required to activate BMP signaling transduction [14,35,36]. Mutant ALK2 did not enhance the basal signaling activity or BMP signaling in BMP receptor type II (BMPR-II) and Activin receptor type-2B (ACVR2B) double-knockout cells [35]. Further studies demonstrated that activation of the ALK2 mutant depends on the kinase activity of receptors but not the ligand-binding activity [35,36]. The phosphorylation level of ALK2 determines the basal activity of mutant ALK2. A study revealed that the threonine residue phosphorylation of T203 in ALK2 plays an essential role in the activation of mutant ALK2 induced by type II receptor [36]. In addition, reductions in the affinity of FKBP12 were also found in other site mutations of ALK2, along with increased basal receptor activity [21,31,37]. Interestingly, mild constitutive activation was generally higher in GS domain mutants than in kinase domain mutants.

Recent studies have revealed that mutant ALK2 activated Smad1/5 signaling in response to Activin A, whereas Activin A acts as an inhibitor of BMP signaling through wild-type ALK2 [6,10,38]. The action of Activin A on the mutant ALK2 and the pathophysiological role of Activin A in FOP is described in more detail in the section below. Given the gain-of-function ALK2 mutants, it is of interest to ask whether an ALK2 mutant is sufficient to drive HO in FOP and whether the pathogenesis is ligand-dependent. A genetic mouse model of FOP has been developed to approach these critical questions [10,39,40]. The ALK2 R206H mutation was introduced into the mouse ALK2 locus to generate conditional ALK2 *^R206H^* knockin mice. The malformation of the first digits of the hind limbs and ectopic bone formation were observed in mice. The mice closely replicated key features of classic FOP in humans. By 6–8 weeks of age, the mice had extensive HO in skeletal muscle. Moreover, histological analyses showed that ectopic bone formation occurred by endochondral ossification. It was also noted that mice formed HO in response to skeletal muscle injury, similar to FOP patients [10,39,40]. In good agreement with this finding, spontaneous and injury-induced HO were also found in mice with conditional expression of ALK2^Q207D^, which is constitutively active ALK2. [41,42]. Therefore, these studies demonstrated that the R206H mutation in ALK2 is necessary and sufficient to induce the classical phenotype in mice, including skeletal malformation and progressive HO, as have been observed in FOP patients.

**Figure 3:**
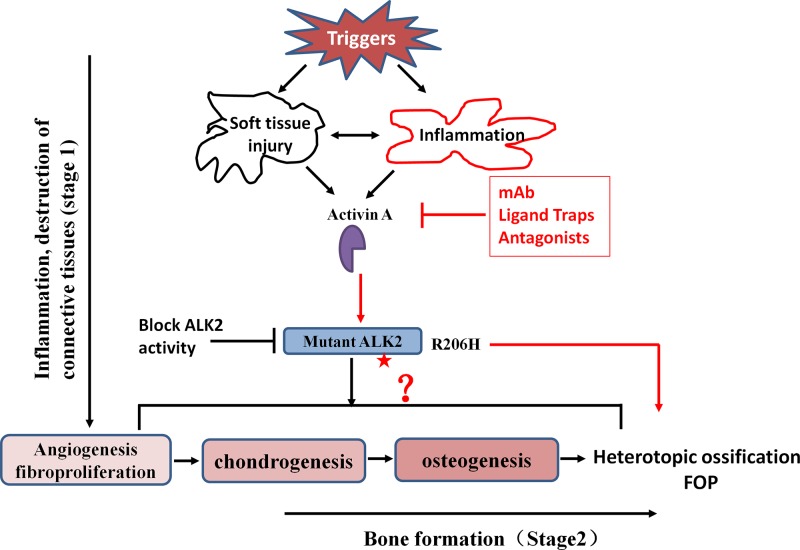
Schematic presentation of Activin A and its involvement in the formation of heterotopic bone in FOP HO formation involves inflammation and the destruction of connective tissues followed by bone formation. After various triggers, such as injury, abundant perivascular lymphocytes are present in damaged tissue. Activin A is secreted by the lymphocytes and induced by inflammation, then activates ALK2 R206H signaling as an osteogenic ligand. Some progenitor cells are recruited and differentiate into chondrocytes and osteoblasts, eventually forming heterotopic bones in soft tissues. However, the previous studies do not indicate the involvement of Activin A in the exact stages of HO formation. In addition, targets and strategies for the prevention and treatment of FOP presents : inhibition of Activin A and block mutant ALK2 activity. See the text for details. The star: classic mutation site of ALK2 R206H.

Since the process of HO is effectively prevented by BMP ligand inhibitors and traps [42,43], it is pertinent to address whether the ALK2 mutation still requires ligands to drive HO in FOP. Hatsell et al. [10] showed that broad-acting BMP ligand inhibitors blocked the HO process in conditional knockin ALK2^R206H^ mice. Moreover, administration of BMP2/4 and Activin A induced the HO process in FOP mice [26,44]. In contrast, using neutralizing antibodies for Activin A and BMP ligand traps effectively inhibited HO formation [10,26]. These studies indicated that the ALK2 R206H mutation caused HO in FOP is somehow dependent on ligands; however, the specific role of BMP ligands in the pathogenesis in FOP remains unexplored. Mutation in ALK2 is crucial for ectopic bone formation in FOP and underlies the pathogenesis of FOP. Thus, a rational therapy for FOP would involve the inhibition of ALK2 function. A number of treatment strategies are being pursued to block mutant ALK2 activity, thereby preventing progressive HO (Figure 3), including small molecule inhibitors [11,42], inhibitory RNA [45,46], antisense oligonucleotide (AON) [47], monoclonal antibodies, and ligand traps [2,48,49]. Presently, these potential approaches that block ALK2 activity are being investigated in cell culture and animal models of HO. For instance, small molecule ALK2 inhibitors, such as dorsomorphin, LDN-193189 and LDN-212854 [50], reduced the ALK2 activity and downstream effectors, and effectively against HO in mouse model of FOP [11,42]. Further extensive studies will be necessary to evaluate the potential efficacy and safety before these treatment strategies can enter into clinical trials for FOP.

## The role of Activin A in FOP: a prominent mediator

### Activin A drives HO in FOP

Activin A is a cytokine and is secreted by innate immune cells, such as neutrophils, monocytes, macrophages, and dendritic cells, and can also be induced by injured soft tissues [51,52]. Activin A generally initiates signaling via type II receptors (ACVR2A, ACVR2B) and type-I receptors (ALK4/7) [53]. Activated receptors phosphorylate Smad2/3 and activate downstream effectors. Activin A can also bind to wild-type ALK2 but cannot activate the phosphorylation of Smad1/5 and inhibit BMP signaling [16,22]. However, recently, two independent studies demonstrated that Activin A abnormally induced phosphorylation of Smad1/5 via mutant ALK2 (R206H) [6,10] (Figure 1). Several cell lines that stably expressed ALK2 R206H gained the capacity to activate Smad1/5 signaling in the presence of Activin A, such as FOP patient-derived induced pluripotent stem cells (FOP-iPSCs), HEK293T cells, human exfoliated deciduous teeth (SHED) cells, and mouse embryonic stem (ES) cells from a genetically derived mouse model of FOP [6,10,27]. Moreover, in ALK2-R206H expressing cells, Activin A significantly stimulated chondrogenesis via BMP signaling [6,26,27]. Activin A enhanced alkaline phosphatase and mineralization of FOP SHEDs and fibro/adipogenic progenitors (FAPs) from a conditional knockin mouse model of FOP [26,27]. Furthermore, blocking the BMP signaling pathway with specific inhibitors diminished the enhanced chondrogenesis differentiation by Activin A. However, whether Activin A can antagonize BMP functions through a common ALK2 R206H mutant receptor is not known.

As the acquired capacity of mutant ALK2 to Activin A was shown to induce Smad1/5 signaling and enhance chondrogenesis differentiation, it is interesting to investigate the physiological role of Activin A in FOP. Taking advantage of FOP-iPSCs and their co-translation with Activin A into the gastrocnemius muscle of nude mice [6], HO formed in the injection site, cartilaginous and calcified cells were observed after 6 weeks. The study indicated that Activin A induced the formation of ectopic bone through mutant ALK2. Consistent with these findings, after tamoxifen activation of ALK2 R206H expression in the conditional knock-in mouse model for FOP (ALK2^R206H^) [10], Activin A was absorbed into collagen sponges and implanted into muscle. The results showed that Activin A initiated bone formation in the implant site. Inhibition of Activin A with anti-Activin A antibodies or Activin A ligand trap ACVR2A-Fc and ACVR2B-Fc have completely blocked spontaneous and trauma-induced HO in the mouse model of FOP. Subsequently, these findings were also confirmed by other studies in an independent mouse model of FOP [26,44,54]. Taken together; these studies demonstrated that Activin A is a prominent mediator for initiating HO in FOP.

### Activin A and its implication in FOP: a potential therapeutic target

The key finding of the prominent role of Activin A in HO opens the door for understanding the crucial molecular basis for FOP. It also provides a very tractable opportunity for blocking Activin A activity as a potential therapeutic target for FOP [2,16,55]. Indeed, there is an ongoing Phase II clinical trial to study the safety and effects of anti-Activin A antibody (REGN2477) in FOP (ClinicalTrials.gov Identifier: NCT03188666). According to its description at ClinicalTrials.gov, the trial aimed to enroll 40 participants, and to study the effect of REGN2477 versus placebo on the change from baseline in HO and the biochemical markers of bone formation in FOP. Additional analysis included assessment of pain due to FOP, the concentrations of total Activin A at baseline and over time, the concentration-time profile and the immunogenicity of REGN2477. To date, no results have been published.

Although studies have shown that Activin-A triggered Smad1/5 signaling and enhanced osteoblast differentiation *in vitro* and induced HO formation *in vivo*, the underlying molecular mechanisms of how a single point mutation in the intracellular region of ALK2 to gain a response to Activin A are largely unexplained. It has been shown that impaired binding of FKBP12 to ALK2 converted the properties of mutant ALK2 [6,7,30,32]. However, subsequent studies excluded the possibility that the only effect of FK506 (an inhibitor of FKBP12) was to enhance Smad1/5 signaling with BMP ligand stimulation. It could not enable wild-type ALK2 gain the capacity to respond to Activin A [10,12]. The other possible explanation was that mutant ALK2 formed stable complexes with type II receptors to convert the ALK2 into an Activin-responsive receptor. Therefore, further studies are required to uncover the molecular mechanism of mutant ALK2 in response to Activin A.

Recently, besides role of Activin A in the transduction of abnormal BMP signaling in initiating HO formation, it also activated mammalian target of rapamycin (mTOR) signaling in FOP-iPSCs [44,56,57]. mTOR signaling is activated during chondrogenic induction. The study also showed that Ectonucleotide Pyrophosphatase/Phosphodiesterase 2 (ENPP2), a known mTOR signaling activator, mediated mTORC1 signaling activation by Activin A. Furthermore, mTOR inhibition by rapamycin potently blocked the enhancement of chondrogenesis *in vitro* and HO formation in the FOP mouse model triggered by Activin A. Rapamycin has been shown to inhibit HO in separate mouse models, including both trauma-induced and genetic HO in FOP [58]. Taken together, these findings suggest that mTOR signaling could be a useful potential therapy for FOP. As the mTOR inhibitor rapamycin has been approved for the prevention of transplant rejection, positive outcomes from these studies will accelerate its translation into a clinical trial for FOP [57,59]. Indeed, an ongoing Phase II/III clinical trial to study the safety and effects of NPC-12T (rapamycin) in FOP is being conducted by Kyoto University in Japan (Identifier: UMIN000028429).

In general, endochondral HO in FOP can be divided into two stages: inflammation and destruction of connective tissues (stage 1) followed by bone formation (stage 2) [5,6]. After soft tissue injuries, abundant perivascular lymphocytes are present in connective tissue, such as skeletal muscle. Lymphocytes infiltrate into damaged tissues, and connective tissue is degraded. Fibroproliferative cells appear and rapidly grow, accomplishing angiogenesis and vascularization, followed by chondrogenesis and osteogenesis, eventually promoting the formation of heterotopic bone in FOP. Numerous growth factors and cytokines, such as Activin A, are involved in this process. It remains to be explored whether the level of Activin A increases locally upon injury or during the entire HO formation process and determines the stages in which Activin A works for (Figure 3). Activin A may act as a biomarker to predict the development of HO.

## Conclusions and perspectives

FOP is a rare genetic disorder and an extremely severe form of HO in humans. Currently, the complete understanding of the molecular mechanism underlying HO is largely unexplained. There are no approved drugs that are effective for FOP. However, over the last decade, basic and clinical research with drug development for FOP has achieved impressive results, leading to a keen interest in FOP since the discovery of ALK2 as the causative gene for FOP. The intense investigation of the molecular mechanism of ALK2 in FOP has clearly indicated the strategy by blockade of ALK2 activation (Figure 3), especially uncovering the prominent and obligatory role of Activin A for activating abnormal BMP signaling via mutant ALK2. The neutralizing antibody or ligand trap to Activin A has shown beneficial effects in inhibiting HO formation. An ongoing clinical trial is examining REGN2477 and is assessing the change from baseline in HO in FOP patients, tolerability, and side effects.

Furthermore, building on recent success in mechanisms of action in FOP, other potential approaches and targets have been proposed, such as targeting inflammatory triggers of flare-ups, targeting connective tissue progenitor cells implicated in HO formation, and targeting the tissue physiologic microenvironmental factors that promote HO [2,48,60]. Some have been shown effective in inhibiting HO in preclinical models of HO and clinical trials [58,61–63]. For instance, Palovarotene, a retinoic acid receptor γ (RARg) agonist, is undergoing a clinical Phase III trial to study its efficacy and safety in FOP (NCT03312634). Greater therapeutic for FOP will be based on proper combination approaches with different targets (i.e., key molecular or cellular targets), mechanisms, and stages. Various approaches may lead to great achievements, with short-term prevention of HO from flare-ups or long-term blockade of the signaling pathway, such as combining anti-Activin antibody with anti-inflammatory agents as an immunosuppressant to evaluate the efficacy, safety, and reduce the side effects.

Genomic and transcriptomic profiles of blood from FOP patients or tissues from mouse models of FOP are providing new insights into the molecular determinants of flare-ups and progressive HO. It is possible to identify the specific role of Activin A in the natural development of flare-ups and the progression of FOP. Characterizing Activin A role in FOP patients will allow for improved disease classification and therapeutic decisions. By performing similar local and systematic analyses at baseline, progress will be made in characterizing the molecular and cellular aspects of the pathophysiology of FOP. For instance, what is the role of Activin A in the interaction between the progenitor cell and immune system cells? If Activin A is secreted by the immune system cells, what about the other cell types, such as skeletal muscle cells, at the site of damaged tissue; what kind of cells will respond to Activin A and give rise to HO?

In conclusion, it needs to continue to explore the mechanism of Activin A in the BMP signaling pathway through mutant ALK2 and its involvement in the pathogenesis of HO in FOP. The discovery of an Activin-mutant ALK2 signaling axis constitutes a robust potential therapeutic target for FOP.

## Abbreviations

ALK2/ACVR1: activin receptor-like kinase 2
BMP: bone morphogenetic protein
ES: Embryonic stem cell
FKBP12: FK506-binding protein 1A
FOP: fibrodysplasia ossificans progressiva
GS domain: glycine-serine-rich domain
HO: heterotopic ossification
iPSC: induced pluripotent stem cell
mTOR: mammalian target of rapamycin
SHED: human exfoliated deciduous teeth
Smad: Signaling mothers agaist decapentaplegic
